# Non-invasive cumulus cell analysis can be applied for oocyte ranking and is useful for countries with legal restrictions on embryo generation or freezing

**DOI:** 10.1371/journal.pone.0297040

**Published:** 2024-01-31

**Authors:** Tom Adriaenssens, Inge Van Vaerenbergh, Lisbet Van Landuyt, Greta Verheyen, Michaël De Brucker, Michel Camus, Peter Platteau, Michel De Vos, Maria Reis, Elien Van Hecke, André Rosenthal, Johan Smitz

**Affiliations:** 1 Fertiga, Brussels, Belgium; 2 Brussels IVF, Universitair Ziekenhuis Brussel, Brussels, Belgium; 3 Follicle Biology Laboratory, Vrije Universiteit Brussel, Brussels, Belgium; Kasr Alainy Medical School, Cairo University, EGYPT

## Abstract

**Research question:**

Can a strategy for scoring oocyte quality, based on cumulus cell (CC) gene expression, prioritize oocytes with the highest implantation potential, while limiting the number of embryos to be processed in culture and the number of supernumerary embryos to be vitrified?

**Design:**

An interventional, blinded, prospective cohort study was retrospectively analyzed. In the original study, patients underwent a fresh Day3 single embryo transfer with embryos ranked based on morphology and CC gene expression (Aurora Test). The additional ranking of the embryos with the Aurora Test resulted in significant higher clinical pregnancy and live birth rates. Now it is investigated if the Aurora Test ranking could be applied to select oocytes. The effect of an Aurora Test based restriction to 2 and 3 2PN or MII oocytes on clinical pregnancy and other outcomes, was analyzed in two subsets of patients with all 2PN (n = 83) or all MII oocytes (n = 45) ranked.

**Results:**

Considering only the top three ranked 2PN oocytes, 95% of the patients would have received a fresh SET on Day3 resulting in 65% clinical pregnancies. This was not different from the pregnancy rate obtained in a strategy using all oocytes but significantly reduced the need for vitrification of supernumerary embryos by 3-fold. Considering only top-ranked MII oocytes gave similar results.

**Conclusions:**

In countries with legal restrictions on freezing of embryos, gene expression of CC can be used for the selective processing of oocytes and would thus decrease the twin pregnancy rate and workload, especially for embryo morphology scoring and transfers as the handling and processing of lower competence oocytes is prevented, while improving the ART outcome.

## Introduction

Legal restrictions on embryo freezing in countries like Germany and Poland and formerly also in Italy [1], limit the number of embryos to be generated and processed in Assisted Reproductive Technologies (ART). The German Embryo Protection Act limits the generation of embryos which are not transferred to the patient. For this reason, the number of oocytes fertilized is limited (e.g. 3 or 5). The Act on Infertility Treatment in Poland allows up to 6 fertilized embryos on a standard basis. With these restrictions the pregnancy chances in fresh transfer cycles are also reduced. This results in a longer time-to-pregnancy for these patients. For example, Italian ART centers reported live birth rates of 19.1% and 14.9% for fresh transfers and cumulative live births of 32.6% and 26.5% for 36- and 36–39-year-old patients, respectively [2]. Cumulus cell (CC) gene expression and its correlation with the oocyte quality has been demonstrated by many groups before (for review: [3, 4]).

Non-invasive oocyte quality scoring based on cumulus cells (CC) gene expression analysis is an appealing non-invasive strategy to prioritize embryos with the highest potential for the fresh transfer cycle and thus enables higher pregnancy rates per transfer [4, 5]. The CC gene expression analysis applied in the current study is the Aurora Test. It is a molecular test analyzing the expression of genes that proved to be predictive for clinical pregnancy in earlier studies [6, 7]. Further extending the use of this predictive test, one can consider restricting the number of oocytes (the higher ranked) to be grown to day 3 or day 5 embryos, as this could reduce the workload in the embryology lab and prevent the generation of supernumerary embryos. This novel strategy would be in line with legislations in Germany, Poland and other countries without compromising the chances for the couple with respect to success rates in the first fresh transfer. In addition, lower ranked oocytes would still be cryopreserved and could be used later if needed. Thus, such an oocyte selection strategy based on a non-invasive testing and ranking method would potentially reduce the time-to-pregnancy for the patient, and very importantly, also reduce work and costs for the ART labs. Fewer embryos would need to be cultured and evaluated, fewer transfer cycles would be required, and fewer supernumerary embryos would need to be frozen.

To evaluate this strategy, a prospective study in which 113 patients had their CC analyzed with the Aurora Test [5] was retrospectively analyzed.

## Methods

In an interventional, assessor-blinded, cohort study, approved by the Ethics Committee of Universitair Ziekenhuis Brussel (Vrije Universiteit Brussel) (BUN143201318000/143201628797) 113 patients planned for fresh Day3 elective single embryo transfer (eSET) signed written informed consent and underwent the Aurora testing on their CC for ranking their embryos. A secondary analysis of 83 patients who had undergone CC-analysis of all their 2PN’s (n = 571 total oocytes) and 45 patients who had undergone CC-analysis of all their MII oocytes (n = 361 total oocytes) was performed in this manuscript. A study flowchart can be found in S1 Fig.

The strategy described in this manuscript requires individual COC denudation. The eventual effect of individual COC denudations with Cumulase and certified denudation/handling pipets (eg: Swemed Vitrolife, EZ-tip Cooper Surgical) has been evaluated on several occasions and no effect on 2PN formation was reported compared to pooled COC denudation [5, 7]. The Aurora Test has been described in length before [5]. In brief, CC of individual denuded oocytes were snap frozen in liquid nitrogen and later applied for total RNA extraction (RNeasy Micro kit, Qiagen The Netherlands). All RNA was reverse transcribed with the iScript cDNA synthesis kit (BioRad, Belgium). cDNA was frozen at − 80°C until further qPCR analysis. With qPCR analysis, the mRNA expression for three specific genes (EFNB2, SASH1, and CAMK1D) was analyzed together with two endogenous control genes, UBC and B2M. The mean of the B2M and UBC expression was used as the normalization factor. All qPCR quantifications (LC480, Roche Diagnostics) were performed in duplicate or triplicate for the samples, and in triplicate for the calibrators and negative controls. The average coefficient of variation was < 0,1 Cp for all assays applied, and negative controls were generated by omitting the enzyme or the RNA in the RT reaction and NTC were added to each qPCR.

From the normalized qPCR expression data for the three specific genes, an embryo/oocyte ranking was created using a mathematical formula (patent WO2014125129A1). The Aurora Test was performed in a specialized molecular lab by people blinded for the eventual fertilization and or development and it took 6 to 8 hours to perform. A CC based ranking was sent to the embryo lab. In the embryo lab good and top-quality embryos were selected based on morphology, and the highest ranked embryo, according to the Aurora Test results, was transferred.

The 520 patients in the control arm underwent a fresh Day3 eSET without the Aurora Test. Patients included in the study had their first or second ART attempt, were younger than 40, had a normal BMI, were stimulated with GnRH Antagonist/HP-hMG, and were scheduled for ICSI and eSET and had ≥2 GQE on Day3. The Aurora Test would not be offered to patients with a poor ovarian response, since there is most likely no choice to be made, and also patients with PCOS or severe male infertility were excluded.

This study revealed a significantly higher clinical pregnancy of 61% in the patients with eSET based on CC ranking applied on good morphology embryos, compared to 29% in the control group with transfer based on embryo morphology alone (weighted generalized linear model, p < 0.0001). Live birth rate was also significantly increased while time-to-pregnancy was significantly reduced with 3 transfer cycles in the CC tested group (Kaplan-Meier, p < 0,0001) [5].

The potential effect of ranking and selecting oocytes, instead of the Day3 cleavage embryos, with a CC based score was retrospectively analyzed in two subsets of patients of the above-mentioned study which had at least four 2PN oocytes on Day1 to ascertain that the proposed strategy could be evaluated. In the first set, 83 patients had all their fertilized oocytes (2PN) CC-analyzed (n = 571). In the second set, 45 patients had all their MII oocytes (n = 361) CC-analyzed and ranked. General patient characteristics are described in S3 Table. Oocytes were ranked only based on the CC test result, while ignoring fertilization and embryo development. The impact of retaining only the (i) top two-ranked oocytes, (ii) the top three-ranked oocytes or (iii) all oocytes on the chance of transfer and clinical pregnancy for these patients was calculated.

Statistical analysis was performed between a strategy where all oocytes were fertilized and cultured (referred to as “All Oocytes” in Table 1) and a strategy where only the top 3 ranked 2PN oocytes, according to the CC gene expression (Aurora Test), are cultured and considered for transfer (referred to as “Top 3” in Table 1). The statistical analysis was the 2-sided Fisher Exact test (p<0.05) using GraphPad Prism V9.5.1.

**Table 1:** Potential effect of restricting the number of 2PN oocytes to grow to embryos, based on the oocyte ranking with the Aurora Test in patients with at least 4 2PN oocytes (n = 83 patients).

	If considering				
	**Top 2 oocytes**	**Top 3 oocytes**	**All oocytes**		
2PN Oocytes considered	166	249	571		
GQE Available Day3	125	177	423	Day 3	
% Patients with a fresh transfer (#)	89% (74)	95% (79^ns^)	100% (83^ns^)		
% Pregnancies/fresh Transfer (#)	65% (48)	65% (51^ns^)	65% (54^ns^)		
Fraction of the GQE frozen (#)	41% (51)	55% (98*)	80% (340*)		
# Of GQE Frozen/Patient with transfer	0,69	1,24	4,10		
# Additional FRET Cycles	7	15	47	Considering only Top ranked oocytes	
# Additional FRET Pregnancies	3	5	16		
% Cumulative Pregnancies/83 patients	61% (51)	67% (56*)	84% (70*)		
% Cumulative Pregnancies considering all available oocytes/83 patients	84% (70)	84% (70)	84% (70)	

GQE: Good or top-quality embryos based on Day3 morphology, FRET: Frozen/Thawed Embryo transfer cycles of single embryos. Statistical analysis is performed between a group mimicking the embryo growth of only the top 3 ranked 2PN oocytes, according to the CC gene expression (Aurora Test) and a group where all oocytes were fertilized and cultured. The all-oocyte group has a high fresh SET pregnancy rate as it also benefited from the Aurora Test that was used to select the top ranked embryo for transfer. *: indicates significant difference, ns: no significant difference with the 2-sided Fisher Exact test p<0.05 using GraphPad Prism V9.5.1.

## Results

Eighty-three of the 113 patients with at least four 2PN oocytes were retained for the current analysis, resulting in 571 2PN oocytes that were analysed and ranked using the Aurora gene expression test. QPCR raw data is available in S4 Table. On Day3, 423 good quality or transferable cleavage embryos (GQE) were obtained. Thus, a mean number (+/-SD) of 6.9±3.0 2PN oocytes and 5.1±3.1 GQE were obtained per patient and the embryologist had finally to choose one out of five GQE for transfer.

Considering all 2PN oocytes and growing only the 249 top three Aurora Test ranked 2PN oocytes from the total number of 571 2PNs, the number of GQE grown to Day3 could have been reduced from 423 to 177 embryos (Table 1). In this group the number of oocytes/embryos in culture is reduced to 44% (249/571) while there would still be 2.24 (177/79) GQE per patient available for transfer. Ninety-five % of the patients (n = 79) would have received a Day3 fresh transfer resulting in a clinical pregnancy rate of 65%.

Applying a strategy of selecting only the top two ranked 2PN oocytes (n = 166) resulted in 125 GQE on Day3 for transfer and reduced the number of supernumerary embryos generated. This is reflected in the lower fraction of good quality embryos frozen 51/125 compared to 98/177 when selecting the top three ranked 2PN oocytes (2-sided Fisher exact p = 0.0143). Using this strategy, a similar high clinical pregnancy rate of 65% would have been achieved, but only 89% of the patients would have received a fresh transfer.

In contrast, if all 571 2PN oocytes would be cultured which is current standard of care at Brussels IVF, all 83 patients would have received a fresh transfer on Day3 and the clinical pregnancy rate for the first fresh transfer would still be 65% when applying the Aurora Test gene expression ranking. Thus, outcome results are statistically not different between standard of care and a strategy pre-selecting the top three 2PN oocytes for embryo culture and transfer (2-sided Fisher Exact test, p = 0.1205 for the number of patients with a fresh transfer, and p>0.9999 for the number of pregnant patients, Table 1 and S2 Table). However, 246 more embryos would have been grown compared to the top 3 selection strategy and 3 times more supernumerary embryos would need to be vitrified (340 compared to 98).

The strategy of restricting the number of 2PN oocytes for further culture to two or three does not limit future options of the patients, as the Aurora Test is only intended for positive selection. Patients not pregnant from the first fresh transfer could still return for a frozen/thawed cycle with the supernumerary embryos or with the frozen 2PNs still available with the effect that the cumulative clinical pregnancy rate calculated per all 83 patients would increase to 84% in the 3 groups (Table 1). The effect of second pregnancies in the same cohort, has not been studied since the return rate of the patients with a child is too limited to allow any further study.

The above-described analysis was repeated on a second subset of patients originating from the same study. This sample set comprised 45 patients with all available MII oocytes analysed (n = 361) and ranked according to the Aurora Test. Due to the retrospective nature of this study, the number of patients available in this sub analysis was limited. As a consequence the statistical power of the non-inferiority claim in this last subgroup was limited. It was however interesting to see how the Aurora Test ranking would perform when CC samples of oocytes that failed to fertilize (not 2PN = 20% = 72/361) were included. Ultimately the results of restricting the number of oocytes to the top 2 or top 3 highest ranked based on the Aurora Test proved to be similar to the results observed in the first analysis (S1 Table).

## Discussion

A selection strategy of competent MII oocytes based on CC gene expression was already proposed in an explorative study [8]. In the current study 3-times as many patients (n = 83) underwent single embryo transfer, and a predefined pregnancy prediction model, the Aurora Test, that combines the expression of 3 predictive genes was used. The current study evaluated the Aurora Test as a predictive tool to optimize the ART procedure by reducing the embryo culture, minimizing the number of supernumerary embryos to be vitrified, assuring a high transfer rate, and ensuring high clinical pregnancy rates for elective single embryo transfer.

This retrospective analysis mimics the selection of the top three or the top two 2PN oocytes for further embryo culture and fresh transfer based on the highest Aurora Test ranking obtained from CC available on Day 0. A top three 2PN selection strategy would reduce the number of 2PN oocytes to be evaluated from 571 to 249 (>2x work reduction), and the number of supernumerary embryos generated on Day3 from 340 to 98 (>3x reduction), respectively. The additional vitrification of 2PN oocytes that is required in the top three 2PN selection strategy is partially compensated by fewer supernumerary embryos that need to be frozen. Overall, this results in approximately 23.5% more freezing work (322 oocytes + 98 embryos) compared to a culture-all oocytes strategy (0 oocytes + 340 embryos).

The selection of the embryo for transfer on Day3 was also done using the Aurora Test score in the 3 groups. This is also beneficial on the workload as it was earlier demonstrated that this resulted in a reduction of 3 transfer cycles compared to a strategy without Aurora Test scoring [5]. The cost of the routine practice with embryo transfer based on morphological selection could be reduced 40% when performing the Aurora Test on all oocytes. The cost of the proposed top three 2PN selection strategy compared to performing the Aurora Test and growing all oocytes would be further reduced by 12%. Most of the savings originate from the reduced number of transfer cycles needed to obtain a live birth.

The strategy of culturing only the three highest ranked 2PN oocytes for subsequent embryo culture and fresh transfer proved to be as efficient in outcome as culturing all 2PN oocytes and resulted in a similar clinical pregnancy rate per transfer and per cycle initiated. Five % of the patients (n = 4) did not receive a transfer but this result is not statistically significant.

Restricting the selection to the top two ranked 2PN oocytes further significantly reduced the number of supernumerary embryos generated per patient, but still yielded a similar high clinical pregnancy rate of 65% per fresh transfer. However, this selection strategy would lead to a lower fresh transfer rate of 89% compared to 100% using all oocytes.

Restricting to the top two ranked 2PN oocytes could be considered in countries with limitations on embryo generation/freezing while allowing the ART lab to adhere to a stricter SET policy.

Similar results were obtained when all MII oocytes were ranked by CC gene expression, and then the top 3 MII was selected for embryo culture and transfer. This sub-analysis comprised 45 patients and 361 MII oocytes of which 289 (80%) yielded 2PN oocytes and 72 did not fertilize. Interestingly, these 20% unfertilized but CC expression ranked oocytes did neither lower the chance of embryo transfer nor the clinical pregnancy rate compared to the using all oocyte strategy. Hence, a combination of (i) CC expression testing of MII oocytes, (ii) vitrifying all the MII oocytes and then (iii) thawing the top 3 ranked MII oocytes for fertilization, embryo culture and transfer would also be a potential strategy. However, while this MII selection strategy promises additional savings in workload, it would likely delay the time-to-pregnancy.

The Aurora Test expression-based selection strategy of the top three ranked or the top two ranked 2PNs resulted in the same high clinical pregnancy rate of 65% for the first fresh Day3 SET compared to using all 2PN oocytes. These outcomes are clearly significantly higher than the currently reported pregnancy rates for fresh Day3 transfers in German ART centres which often are double embryo transfers (DET). In Germany, clinical pregnancy rates reported in 2019 were 39.9%, 39.2% and 30.6% for <29-, 30-34- or 35–39-year-old ICSI patients, respectively, with a mean of 1.7 embryos transferred on Day3 or 5 (DIR Annual 2019). Two approaches are currently used in German ART centres: (i) the German conservative approach in which three 2PN oocytes are retained followed by a SET or DET on Day3 or (ii) the more liberal “German Middle Way” strategy which attempts to improve treatment results by culturing more than three embryos and selecting those with the greatest potential viability at the blastocyst stage. But results are still in the same range: 31% clinical pregnancy rate for the conservative approach (1.96+/-0.62 embryos transferred [9]) and 35.8% or 42.6% clinical pregnancy rate for the German Middle Way approach followed by SET or DET cycles [10]. The DET cycles resulted in 28.2% multiple pregnancies.

The ranking and selection of 2PN or MII oocytes based on non-invasive CC gene expression thus proves beneficial in ART centers with legal restrictions on freezing embryos at Day3 or Day5, because two times more patients would be pregnant from the fresh Day3 SET. Furthermore, our strategy is coupled with a single embryo transfer (eSET) which reduces the chances of twin pregnancies and avoids potential neonatal and postnatal complications linked with it.

In addition, the oocyte selection strategy based on the Aurora Test could also be applied in countries without legal restrictions on embryo freezing with savings on workload in the embryology lab, especially for embryo morphology scoring and transfers as the handling and processing of lower competence oocytes is prevented. However, this new oocyte selection approach has two limitations: (i) it requires individual oocyte denudation and (ii) expertise in oocyte freezing. Individual oocyte denudation takes only 15 minutes longer compared to pooled denudation and oocyte vitrification is done in many ART centres successfully. However, poor oocyte survival after cryopreservation might compromise the success of the strategy described here. The current oocyte selection analysis could also be useful in oocyte donation cycles and/or fertility preservation cycles. It could even be associated with AI for a standardized morphological assessment.

This new oocyte selection strategy based on CC gene expression merits validation in a prospective clinical study.

## Supporting information

S1 Table Potential effect of restricting the number of MII oocytes to inject and to grow to embryos, based on the oocyte ranking with the Aurora Test in patients with at least 4 2PN oocytes (n = 45 patients). GQE: Good or top-quality embryos based on day3 morphology, FRET: Frozen/Thawed Embryo transfer cycles of single embryos. Statistical analysis is performed between a group mimicking the embryo growth of only the top 3 ranked MII oocytes, according to the CC gene expression (Aurora Test) and a group where all oocytes were fertilised and cultured. *: indicates significant difference, ns: no significant difference with the 2-sided Fisher Exact test p<0.05.
(DOCX)

S2 Table Raw data used to calculate the statistical significances stated in the result section. We compare the strategy using only the TOP3 ranked oocytes to the strategy using all embryos A) % Patients with fresh transfer B) %Pregnancies/ fresh transfer C) Fraction of the GQE frozen D) Cumulative pregnancies/83 patients E) Cumulative pregnancies considering all available oocytes /83 patients.
(XLSX)

S3 Table Patient characteristics of the fresh ICSI cycle in Aurora Test patients. Patient characteristics are described of Aurora Test patients with 2PN oocytes ranked (n = 83) and Aurora Test patients with MII oocytes ranked (n = 45).
(XLSX)

S4 Table Raw data qPCR. Raw data of qPCR analysis (mean Cp values from triplicates) for UBC, B2M, EFNB2, SASH1 and CAMK1D (n = 83 patients).
(XLSX)

S1 Fig Study flowchart. Evaluate the effect of an Aurora Test based restriction to 2 (Top2) and 3 (Top3) 2PN or MII oocytes on clinical pregnancy and other outcomes in two subsets of patients with all 2PN (n = 83) or all MII oocytes (n = 45) Aurora Test ranked.
(TIF)
