# Supplementary material for: Non-invasive cumulus cell analysis can be applied for oocyte ranking and is useful for countries with legal restrictions on embryo generation or freezing
Source: PLoS One. 2024 Jan 31;19(1):e0297040. doi: 10.1371/journal.pone.0297040 (PMC10830053; doi:10.1371/journal.pone.0297040)
Supplement: S1 Table — GQE: Good or top-quality embryos based on day3 morphology, FRET: Frozen/Thawed Embryo transfer cycles of single embryos. Statistical analysis is performed between a group mimicking the embryo growth of only the top 3 ranked MII oocytes, according to the CC gene expression (Aurora Test) and a group where all oocytes were fertilised and cultured. *: indicates significant difference, ns: no significant difference with the 2-sided Fisher Exact test p<0.05. (DOCX) [file pone.0297040.s001.docx]

|  | **If considering** | | |  | |
| --- | --- | --- | --- | --- | --- |
|  | **Top 2 oocytes** | **Top 3 oocytes** | **All oocytes** |  | |
| MII Oocytes considered | 90 | 135 | 361 |  | |
| GQE Available Day3 | 62 | 89 | 216 | Day 3 | |
| % Patients with a fresh transfer (#) | 89% (40) | 93%^ns^ (42) | 100%^ns^ (45) |  |  |
| % Pregnancies/fresh Transfer (#) | 60% (24) | 60%^ns^ (25) | 60%^ns^ (27) |  |  |
| Fraction of the GQE frozen (#) | 35% (22) | 53%^*^ (47) | 79%^*^ (171) |  |  |
| # Of GQE Frozen/Patient with transfer | 0,55 | 1,12 | 3,80 |  |  |
| # Additional FRET Cycles | 1 | 6 | 29 | Considering only Top raked oocytes | |
| # Additional FRET Pregnancies | 1 | 2 | 8 |  |  |
| % Cumulative Pregnancies/45 patients | 56% (25) | 60%^ns^ (27) | 78%^ns^ (35) |  |  |
| % Cumulative Pregnancies considering all available oocytes/45 patients | 78% (35) | 78% (35) | 78% (35) |  |  |
