# Supplementary figures and images for: Non-invasive cumulus cell analysis can be applied for oocyte ranking and is useful for countries with legal restrictions on embryo generation or freezing
Source: PLoS One. 2024 Jan 31;19(1):e0297040. doi: 10.1371/journal.pone.0297040 (PMC10830053; doi:10.1371/journal.pone.0297040)

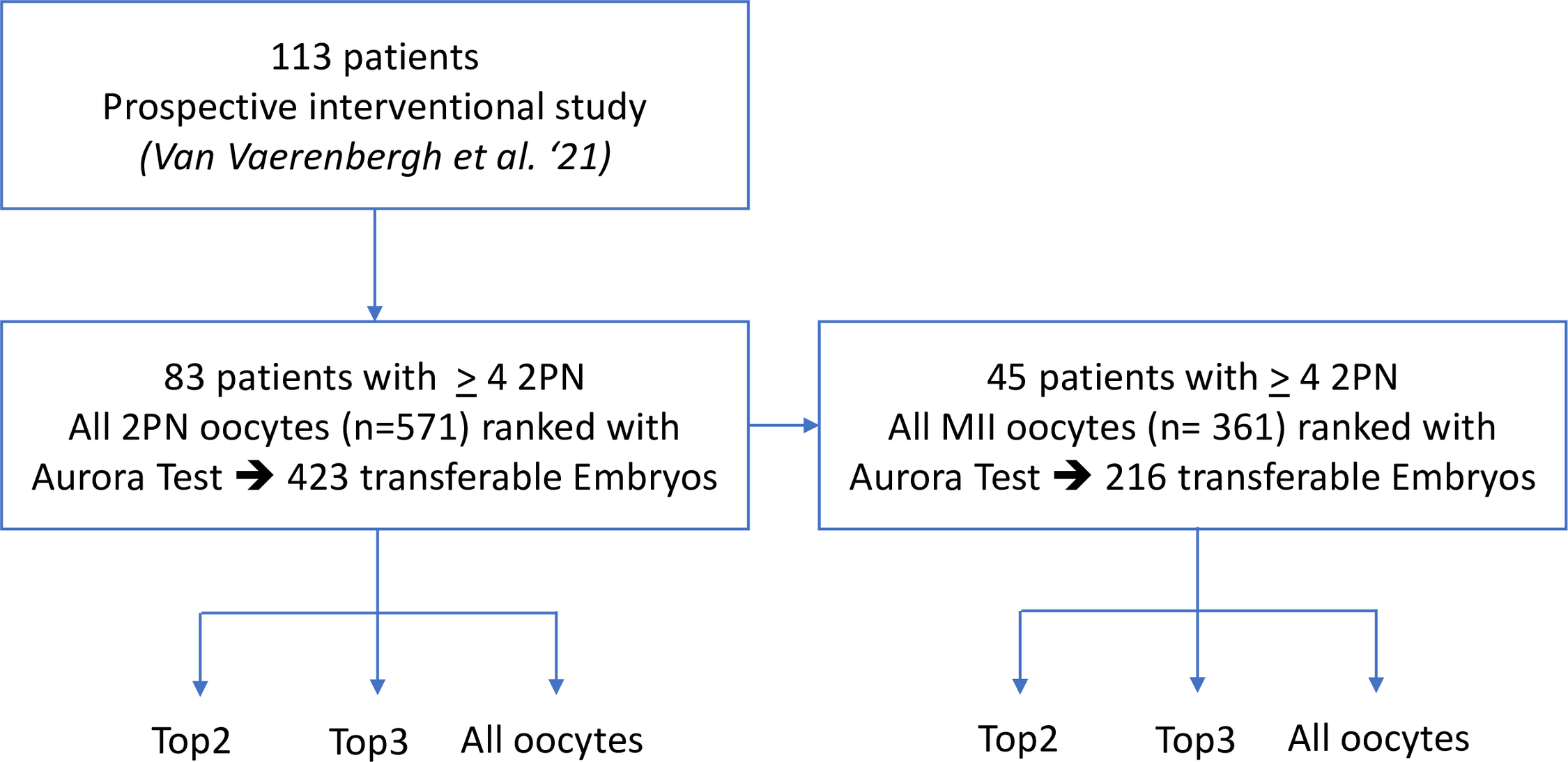

Supplement: S1 Fig — Evaluate the effect of an Aurora Test based restriction to 2 (Top2) and 3 (Top3) 2PN or MII oocytes on clinical pregnancy and other outcomes in two subsets of patients with all 2PN (n = 83) or all MII oocytes (n = 45) Aurora Test ranked. (TIF) [file pone.0297040.s005.tif]
